# Cases of Retained Placenta

**Published:** 1848-01

**Authors:** W. L. Sutton

**Affiliations:** Georgetown, Ky.


### SELECTIONS

FROM

### AMERICAN AND FOREIGN JOURNALS.

---

*Cases of Retained Placenta.* By W. L. Sutton, M D. Georgetown, Ky. Case I.—*Abortion.—Placenta Retained.—No ill Effects.* On 1st September, 1822, Mrs. S. had an abortion, having menstruated about the middle of May, it being the only time since the birth of a child. The fœtus was expelled, but the placenta retained. The umbilical cord remained attached to placenta for about twenty-four hours, then separated. No fetor or offensive discharge followed. The woman recovered her health in the usual time, no unpleasant effects appearing at any time; Nor was any thing which could have been suspected to be the secundines, at any time observed. The woman lived many years, and bore four children.

Case II.—*Abortion.—Placenta Retained.—Subsequent Hemorrhage and Death.* Lucy, aborted on 10th May, 1840, being three months pregnant; the fœtus expelled, but no secundines. She seemed to improve about as well as usual after such accidents, and had returned to her employer, and been engaged in her daily work, when on 15th June she was seized with a violent flooding. It was supposed she lost a gallon of blood in a very short time, and a great deal subsequently. I saw her on 17th, when the hemorrhage had nearly ceased, but she was stupid; great paleness of the mucous membrane of the mouth; paralysis of the lower limbs; pulse rather full, but irritable. Continued to sink until death, which took place on the 21st. Stupor, and paralysis of the lower limbs and of the bladder, continued till death.

Case III.—*Abortion.—No Secundines Found.—Subsequent*

*Hemorrhage.* August 15th, 1847, I was desired to visit Mrs. C., who I found had lost a good deal of blood from the uterus; was doubtful as to pregnancy. Upon examining the coagula, found a fœtus apparently of about two months, which corresponded with her history of her menstruation. No secundines could be found. 17th. The hemorrhage, which had nearly ceased from the expulsion of the fœtus, recurred very freely, and continued for some hours, and then abated. I examined a considerable quantity of the coagula, but could detect no placenta; but I had not an opportunity to examine all the coagula. As the hemorrhage had subsided, and as nothing could be felt at the mouth of the uterus, I felt justified in hoping all had passed. She went on very much as is common after abortions except that a slight discharge continued, until 3d September, when an alarming amount of hemorrhage again took place. Still nothing was to be found at the os uteri. The sponge, impregnated with vinegar, was used, and powders of lead, ipecac. and opium administered; which seemed to arrest the flooding. When removed, the sponge had a very offensive smell, and was colored black, which could not be washed off. A small, but very offensive discharge continued about a week, for which injections of chamomile tea, with a particle of lime infused, were used.

*Remarks.* These cases have caused considerable reflection in my mind. It would seem, from what I have read upon the subject, that the retention of the secundines, at an early period of pregnancy, need not give rise to any apprehension of danger. Thus, Rigby (System of Midwifery, p. 359), says:—"Cases of abortion have occasionally been observed, where the embryo has escaped, but the secundines never come away, although the discharges, &c., have been watched with the greatest attention. After a time, the menses have returned, the patient has again become pregnant, and has passed through her labor at the full term, without anything unusual occurring." Nothing is intimated that unpleasant consequences ever follow. It is true, that a case is mentioned in the American Journal of Medical Sciences, v. iv., p. 511, in which it is said, "the lady remained in indifferent health for three months," when the secundines were expelled. But I am not aware of any case on record in which serious and alarming symptoms have supervened. Is such the experience of the profession? or have cases been lost sight of, and considered as having done well, because nothing more was heard of them? Have I been less fortunate than others? or am I wrong in having attributed the hemorrhage which occurred in two out of three cases, which I have seen, to the retention of the placenta? I have always supposed that the hemorrhage and death in case 2d was occasioned by the retention, although the woman was thought, and considered herself, well, and had returned to her ordinary labor. I am every way satisfied in my own mind, that the hemorrhage in the 3d case was owing to a retention, proved sufficiently, I think, by the offensiveness of the discharge, and the discoloration of the sponge used as a tampon. Therefore it appears to me that the condition of the woman, with retained secundines, even at an early month, is not so safe as is supposed by many. How is this state of things to be obviated? Truly I do not know. No one, I presume, would feel authorised to poke a hook into the uterus, with no guide to direct him; and the cavity of the uterus is too small to admit of any manual operations. The only alternative which I perceive is to use the ergot. This may sometimes succeed, but I know it to have failed.

Case IV.—*Protracted Labor.—Retained Placenta.—Hemorrhage.* Mrs. C., the subject of the last case, was delivered of a large child, after a tedious labor, at 3½ o'clock, P.M, October 17th, 1834. The placenta not coming readily, and considerable hemorrhage existing, I used, for want of ergot, acet. plumbi, ipecac. and opi., with frictions to abdomen, warmth to feet, and volatiles. At 6 o'clock, nothing having been gained, I introduced my hand, and found a partially separated placenta, adherent to the fundus uteri, it being uncontracted. By pressing moderately on the adherent portion of the placenta, and by insinuating the points of the fingers under the free edges, I effected a separation. After moving the placenta about for some time, rubbing it against the walls of the uterus, I extracted it. Hemorrhage continued free for some time; but eventually subsided under the use of frictions and the pills of lead, ipecac. and opium.

*Remarks.* This is the only case of adherent placenta which has occurred to me in a practice of twenty-eight years; if we except one which I found attached to an inverted uterus. I was guilty of mal-practice in this case, in separating and extracting the placenta before the uterus had properly contracted; and thereby exposed the woman to unnecessary hazard from hemorrhage. It is true, that I kept my hand in the uterus some time, and employed friction on the surface of the uterus; but I should have continued it until contraction took place.

Case V.—*Placenta Retained by Premature Contraction of the Os Uteri.* June 15, 1827, was desired to see Mrs. R., who had been delivered of her first child fifteen hours—placenta retained. Upon inquiry, I learned that the placenta had been felt at the mouth of the uterus, soon after the birth of the child, but would not come away—that the umbilical cord had given way during the efforts at extraction—that a loop had been fixed over the remaining portion. Upon examination, I found it just so. The insertion of the cord into the placenta offered directly at the middle of os uteri—the mouth itself very much contracted. By gently dilating it, I was enabled to get two fingers up by the side of the placenta, and fixing them in it, turn one edge down and extract it. The woman declared that the pain of this operation was equal to that of labor.

Case VI.—*Placenta Retained by Premature Contraction of Os Uteri.* August 26, 1833, I saw a negro woman under the care of Dr. D., who had been delivered of her first child three days, the placenta retained. I found the funnel-shaped portion at the os uteri; no lochial discharge, but a very offensive fetor; slightly feverish, but no pain. A scruple of ergot was given, which produced considerable pains, which, however, produced no effect on the placenta. Waiting several hours, and finding matters no better, I introduced my hand into the uterus, and hooking my forefinger into the body of the placenta and embracing it firmly with the others, I succeeded in extracting it. This required considerable time as the mouth and body of the uterus were firmly contracted. Some idea of the firmness of the contraction may be formed, when I state, that two months elapsed before my hand had recovered entirely from the compression which it underwent whilst in the uterus. It is perhaps proper to remark, that I was in delicate health at the time, which may have prevented a more speedy abatement of these effects.

*Remarks.* It has been a rule in my obstetrical practice (inculcated by Prof. Hall, of the University of Maryland), as soon as I have disposed of the child, to take the chord in my left hand as a director, and run the finger of my right along it to the os uteri; if I find the funnel-shaped portion of the placenta there, insert a finger and hook it over the edge and bring it down and deliver it at once. Pursuing this course, I have not had, perhaps, a dozen placentæ expelled by the uterus exclusively, during my practice. From this practice I have at no time seen any inconvenience. On the contrary, it relieves the woman from that state of dread and perturbation, which is almost sure to take place if the placenta is not removed in a short time after the birth of the child. I do not know that this course was admissible in the last two cases; but from the statement of the genteman in attendance upon the first, I am induced to believe it would have been in that case.

In case 6th, the ergot failed to do any good, although it evidently produced a good deal of suffering. That it would have failed, if repeatedly used, I cannot say. Dr. Dewees speaks strongly of his confidence in its powers. To be sure, that confidence was founded on analogy of its action in expelling the secundines after abortion. Dr. Porcher, however, of South Carolina (American Journal Medical Sciences, v. x., p. 391), shows that it cannot *always* be depended on. Dr. Jackson, too, of Northumberland, Pa. (Medical Recorder, v. xv., p. 362), argues against not only the efficacy, but the safety of the article in these cases. Let us look into the state of the uterus, and consider the effects of the medicine. The os uteri was firmly contracted; of course it must be dilated before the placenta could be expelled. Will the ergot cause that dilatation ? I presume most men will say, that although it may produce this effect, yet we cannot calculate upon it with certainty—that it will frequently fail. If it does not produce that effect, it adds to the difficulty, by diminishing the cavity of the uterus, and also of its mouth, in consequence of producing a general contraction of the uterine fibres. If the intention is to introduce the hand if the ergot fail, we ought to change our purpose, and introduce the hand in the first instance; because the use of the ergot will inevitably increase the difficulty of the manual operation. If this view is correct, Dr. Dewees' advice to use ergot first is incorrect; and I doubt not but that I had much greater difficulty than I should otherwise have had, in consequence of following his directions.

Was it necessary to introduce the whole hand ? This may be considered doubtful. It will be recollected that no one could tell whether the placenta would be found detached. In fact, after my hand was introduced, I could not say with assurance that it was detached, it was embraced with such firmness by the uterus. Again, it is doubtful whether the motions necessary to force a finger into the placenta and extract it, could have been effected with the hand mainly without. At any rate, I am satisfied that the aid of the other fingers and thumb was very serviceable in extraction.

Ought the case to have been left to nature? This is a grave question, which will be answered differently by different persons. Some, in view of the fact that the woman did as well as she possibly could under any circumstances, will say that the treatment pursued was the best. Others, in view of the fact that many cases, left entirely to nature, do very well, the placenta in some cases being expelled at an indefinite period, in other cases not at all, will say that the woman was subjected to great and unnecessary pain. That many cases of retention have done well, is true; that many have been followed by unpleasant and fatal consequences, is likewise true. It is also true, that a woman is always uneasy and restless until the placenta is removed. Again, it is true, that no man can say of any case of retention, whether, if left to itself, it will terminate favorably or unfavorably. The idea seems to be that if air be excluded from the placenta, putrefaction will not take place and evil will be prevented. Granting this to be true, the same difficulty remains; no man can tell whether air will find access or not. Neither have we any means to prevent such access. Yet does it seem to me, that there is little probability of air finding its way up the vagina and into the uterus. I am therefore inclined to suspect that putrefaction is owing to some other cause.

In volume xxvi. of the American Journal of Medical Sciences is a very valuable paper by Dr. E. Warren, of Boston, on retained placenta, in which, however, he insists rather strongly upon the powers of nature. That she will, in many instances, accomplish wonders, is no less true than fortunate for mankind. If we had any means of judging when she would show her power, we should know when to trust her. Our profession is, I think, too prone to exhibit successful cases to the world, and keep the unfortunate ones back.* Hence we have but little means of forming a true estimate of the number of fatal cases. It seems to me, that Dr. W. has worded one of his sentences, so as to make an erroneous impression, and that that impression is likely to do mischief. Speaking of Dr. Hunter's practice of leaving the placenta to nature, he says—"Finally, some unfavorable cases occurred, and the practice was changed." This would, I think, convey the idea to most persons, that the number of cases was small. But Dr. H. was not a man to be turned from a course, which he considered right, by trifling considerations. Again, I have seen it stated, that by pursuing his course, he lost a certain number of ladies of rank in one year. I do not now remember the number, but I do remember that I thought it was quite enough to make him pause and consider his ground.

I do not wish to convey the impression, that the placenta is

*This reflection is not applied or applicable to Dr. W., so far as I know.

to be speedily removed at all hazards. I should by no means be willing to kill a man with opium, to prevent him from dying with colic. What I advocate is, that we use all due means to remove the placenta with safety to the mother. What these means are, and when they have been used, will be differently estimated by different persons of equal respectability in the profession. As a general rule, he who is best informed as to the success of means used by others, and best qualified to judge of the powers of his patient's constitution in a given case, will be most apt to do right.

Case VII.—*Quick Labor.—Hour-glass Contraction.* Mrs. P. was confined August 26th, 1840. The regular attendant being out of the place, at length I was requested to visit her. The child had been born about two hours before my arrival. Upon taking hold of the cord, and running my finger up it, I encountered an hour-glass contraction. Gave seventy-five drops of laudanum, and waited about three quarters of an hour, when I found the stricture to yield readily and permit the delivery of the placenta.

Case VIII.—*Lingering Labor.—Hour-glass Contraction.* March 3d, I saw Mrs. W. in consultation with Dr. C. She had been in labor about twelve hours. The os uteri being pretty well dilated, and the pains trifling and unavailing, we concluded to give ergot. After giving three portions, the pains became more frequent, and although very short, began to produce an effect upon the progress of labor. In about an hour she was delivered of a dead child. The placenta not presenting itself in due time, Dr. C. introduced his hand, and found an hour-glass contraction. Gave half a grain of sulph. morphia, and waited half an hour. After this interval, Dr. C. again introduced his hand, and, with slight trouble, dilated the stricture and delivered the placenta.

Case IX.—*Lingering Labor.—Hour-glass Contraction.* April 28, 1845, saw Mrs. E. with Dr. C. She had a lingering labor, for which it was considered necessary to give ergot. There being some delay in the expulsion of the placenta, Dr. C. introduced his hand and found an hour-glass contraction. We gave half a grain of morphia, and waited half an hour, when placenta was found at the mouth of the uterus and readily extracted.

Case X.—*Lingering Labor, with considerable Flooding at the commencement.—Hour-glass Contraction.* Sept. 20th, 1847, I was called to attend Judy, a negro woman, exceedingly fleshy, in labor with her thirteenth child. The membranes were represented as having given way an hour and a half before my arrival; before and after which, there had been considerable hemorrhage. There was also some after my arrival, but it soon ceased without interference. Perhaps a pint and a a half had been lost altogether. At this time, 5, P. M., the pains were trifling and at long intervals, and were said to have been in the same condition all day. The os uteri, however, was considerably dilated, but no part of the child could be felt in the common examination. At 10, the mouth was fully dilated, and the head advancing in the pelvis. At 12, the head presented at the lower strait. About this time the pains abated very much, and scarcely made any impression on the head, that little being lost at the end of the pain. Teas, &c., failing to excite contractions, at 2, A. M., ergot was given, and the child born about 3, A: M. In about half an hour, the placenta remaining beyond the reach of the finger, I placed my left hand upon the abdomen, and found, in the epigastrium, a tumor about as large as an uterus well contracted after the expulsion of the placenta, with a hard ridge extending to the pubis. I at once suspected an hour-glass contraction, and, upon introducing my hand, found it so. I gave five-eighths of a grain of sulph. morphia, intending to wait an hour. At the expiration of the time, found the patient asleep; which continuing, an hour and a half elapsed before I undertook the delivery, when I found the placenta loose, and lying at the mouth of the uterus.

*Remarks.* What is the cause of the hour-glass contraction? "Dr. Douglass, of Dublin, considers this condition as arising from some irritation near the mouth of this organ," and "concludes that whenever it does occur, it is produced by mismanagement." This opinion, by having been repeated by a dozen or so of the most eminent accoucheurs, has acquired much authority; yet it is not a particle more true now, than it was when first uttered. If true, how did it happen that I found that condition in the seventh case, when I suspected nothing, and expected nothing but to bring away the placenta as after ordinary labor? How came Dr. C. to find it so in cases 8 and 9? It is true that I cannot positively assert that he committed no indiscretion upon the os uteri; but I do know that he is cautious and prudent, and therefore I am not at all disposed to believe that he did. In the 7th and 10th cases, if tightening the cord and running the finger up it to ascertain the presence or absence of the placenta at the os uteri be mismanagement, then I perpetrated it in both cases; otherwise I did not. If it was mismanagement, how are we ever to manage aright; unless indeed we trust the placenta to the powers of nature, without even attempting to ascertain what is going on?

I have heard ergot charged with producing this state. It will be observed, that in three of the four cases detailed, it was administered. But the cases in which this condition is apt to occur, are precisely those in which we are most apt to give ergot. When, therefore, we reflect that this contraction takes place in many cases of tedious labor, where no ergot is given; and, on the other hand, it is given in many cases of tedious labor with the most beneficial results, without any untoward effects, either immediately or subsequently, we shall pause before we lay this particular evil at its door. We can say that this affection consists in an irregular, a spasmodic contraction of the fibres of the uterus; but we had as well not say upon what that spasmodic contraction depends, until we know.

Is there any particular portion of the uterus to which this action is confined? Authors would seem to confine it to the neck of the uterus; at any rate, not above the commencement of the body. In case 7th, it was but a short way above the os uteri; so little, indeed, that there could scarcely be said to be a lower chamber. In case 10th, I should place it much above the union of the body and neck. I introduced nearly the one half of my fore-arm into the vagina; so that at least my wrist and hand must have been within the uterus. There was ample room to move my hand about in the lower chamber; and with my fingers extended, I ascertained the existence of the stricture, which appeared to extend directly across the body, embracing tightly the cord and a small portion of the placenta. Could a contraction of the lower portion of the body afford such room in the lower chamber? I apprehend not. I did not introduce my hand in either the 8th or 9th case;but from Dr. C.'s account, I consider them as very parallel to the 10th. He indeed is disposed to locate the stricture very close to the fundus.

I do not know that any writer or teacher advocates the use of opium in this affection; and yet it is one in which we should expect it to be beneficial. If there was a spasmodic contraction of any other muscle, opium is the very thing which would present itself to my mind. Again, so far as my experience goes, the parts concerned are exceedingly tender; so much so, that the bare examination necessary to ascertain the existence of the contraction, inflicts much suffering. What torture a woman must suffer, who undergoes the dilatation of parts so excessively irritable, without a previous anodyne, no man can tell, even after witnessing the operation. On the other hand, in two of the four cases, when the attempt to remove the placenta was about to be made, the placenta was found lying loose at the mouth of the uterus. Is there fear that an anodyne will prevent the tonic contraction of the uterus? This last is much more apt to take place when the irregular contraction is removed, than during its continuance. In none of the cases was there any hemorrhage either before or after the administration of the ergot; neither was there any bad effect whatever from its use. If, then, the use of opium is safe as a means of facilitating the dilatatiou of the stricture, or of preventing altogether the necessity of the operation, performing this operation without a previous anodyne must be considered downright cruelty.

Whether the placenta was attached in any one instance when the anodyne was administered, or not, of course is not known.—*Boston Med. and Surg. Jour.*

